# Are we missing ‘previously treated’ smear-positive pulmonary tuberculosis under programme settings in India? A cross-sectional study

**DOI:** 10.12688/f1000research.18353.2

**Published:** 2019-05-16

**Authors:** Hemant Deepak Shewade, Vivek Gupta, Srinath Satyanarayana, Atul Kharate, Lakshmi Murali, Madhav Deshpande, Naresh Kumar, Prabhat Pandey, U N Bajpai, Jaya Prasad Tripathy, Soundappan Kathirvel, Sripriya Pandurangan, Subrat Mohanty, Vaibhav Haribhau Ghule, Karuna D Sagili, Banuru Muralidhara Prasad, Sudhi Nath, Priyanka Singh, Kamlesh Singh, Gurukartick Jayaraman, P Rajeswaran, Binod Kumar Srivastava, Moumita Biswas, Gayadhar Mallick, Om Prakash Bera, A James Jeyakumar Jaisingh, Ali Jafar Naqvi, Prafulla Verma, Mohammed Salauddin Ansari, Prafulla C Mishra, G Sumesh, Sanjeeb Barik, Vijesh Mathew, Manas Ranjan Singh Lohar, Chandrashekhar S Gaurkhede, Ganesh Parate, Sharifa Yasin Bale, Ishwar Koli, Ashwin Kumar Bharadwaj, G Venkatraman, K Sathiyanarayanan, Jinesh Lal, Ashwini Kumar Sharma, Ajay MV Kumar, Sarabjit S Chadha

**Affiliations:** 1 International Union Against Tuberculosis and Lung Disease (The Union), Paris, 75006, France; 2 The Union South East Asia, New Delhi, 110016, India; 3 All India Institute of Medical Sciences (AIIMS), New Delhi, 110029, India; 4 State TB Cell, Department of Health & Family Welfare, Government of Madhya Pradesh, Bhopal, 462004, India; 5 State TB Cell, Department of Health & Family Welfare, Government of Tamil Nadu, Chennai, 600006, India; 6 State TB Cell, Department of Health & Family Welfare, Government of Chattisgarh, Raipur, 492002, India; 7 State TB Cell, Department of Health & Family Welfare, Government of Punjab, Chandigarh, 160022, India; 8 Voluntary Health Association of India (VHAI), New Delhi, 110016, India; 9 Post Graduate Institute of Medical Education and Research (PGIMER), Chandigarh, 160012, India; 10 MAMTA Health Institute for Mother and Child, New Delhi, 110048, India; 11 Catholic Health Association of India (CHAI), Secunderabad, 500009, India; 12 Resource Group for Education & Advocacy for Community Health (REACH), Chennai, 600014, India; 13 Population Services International (PSI), New Delhi, 110019, India; 14 Catholic Bishops’ Conference of India-Coalition for AIDS and Related Diseases(CBCI-CARD), New Delhi, 110001, India; 15 Emmanuel Hospital Association (EHA), New Delhi, 110019, India; 16 Yenepoya Medical College, Yenepoya (Deemed to be University), Mangaluru, 575018, India

**Keywords:** Tuberculosis/classification, Previously treated TB, New TB, Recurrent TB, Vulnerable populations

## Abstract

**Background: **In 2007, a field observation from India reported 11% misclassification among ‘new’ patients registered under the revised national tuberculosis (TB) control programme. Ten years down the line, it is important to know what proportion of newly registered patients has a past history of TB treatment for at least one month (henceforth called ‘misclassification’).

**Methods:** A study was conducted among new smear-positive pulmonary TB patients registered between March 2016 and February 2017 in 18 randomly selected districts to determine the effectiveness of an active case-finding strategy in marginalised and vulnerable populations. We included all patients detected through active case-finding. An equal number of randomly selected patients registered through passive case-finding from marginalised and vulnerable populations in the same districts were included. Before enrolment, we enquired about any history of previous TB treatment through interviews.

**Results:** Of 629 patients, we interviewed 521, of whom, 11% (n=56) had past history of TB treatment (public or private) for at least a month: 13% (34/268) among the active case-finding group and 9% (22/253) among the passive case-finding group (p=0.18). No factors were found to be significantly associated with misclassification.

**Conclusion:** Around one in every ten patients registered as ‘new’ had previous history of TB treatment. Corrective measures need to be implemented, followed by monitoring of any change in the proportion of ‘previously treated’ patients among all registered patients treated under the programme at national level.

## Introduction

India has the highest tuberculosis (TB) burden in the world. The annual estimated TB incidence and deaths is 2.7 million and 0.4 million, respectively ^1^. Of the patients receiving treatment under its revised national tuberculosis control programme (RNTCP), the proportion of ‘previously treated’ patients (received anti-TB drugs in the past for one month or more) was 19% in 2016 and 15% in 2017 ^2, 3^. The national anti-tuberculosis drug resistance (2014–16) survey shows that ‘previously treated’ TB patients have four times higher prevalence of multidrug-resistant TB (MDR-TB) when compared to new patients (11.6% versus 2.8%) ^4^.

In 2007, Atre *et al.* ^5^ reported 11% misclassification among ‘new’ patients registered under the RNTCP. It is important to know how the programme is faring 10 years down the line. This study was carried out as a part of a larger study among new smear-positive pulmonary TB patients to determine the effectiveness of a community-based active case-finding (ACF) strategy when compared to passive case-finding (PCF) in 18 randomly selected districts of India ^6, 7^. The ACF strategy was conducted as part of Project *Axshya* (meaning ‘free of TB’) whose focus was to increase detection of new smear-positive pulmonary TB patients among marginalised and vulnerable populations. Before enrolling the newly registered TB patients (both ACF and PCF patients) into our study, we enquired about their history of previous treatment. This provided us with a unique opportunity to document the proportion of newly registered smear-positive pulmonary TB patients that had previous history of TB treatment and were therefore misclassified (henceforth called ‘misclassification’).

## Methods

### Study design and participants

This was a cross-sectional study involving new smear-positive pulmonary TB patients (≥ 15 y) from marginalised and vulnerable populations that were registered for treatment under the RNTCP in India between March 2016 and February 2017.

### Setting

National TB programme (2016–17): India’s RNTCP infrastructure included national, state, district and sub-district level administrative units (one for 250 000 to 500 000 population) and designated microscopy centres for sputum smear microscopy ^8^. Before starting TB treatment, the medical officer in the health facility classified the patients as ‘new’ or ‘previously treated’.

During the study period (March 2016 to February 2017), new patients received two months of Isoniazid, Rifampicin, Pyrazinamide and Ethambutol followed by four months of Isoniazid, Rifampicin and Ethambutol. ‘Previously treated’ patients received two months of Isoniazid, Rifampicin, Pyrazinamide, Ethambutol and Streptomycin, one month of Isoniazid, Rifampicin, Pyrazinamide and Ethambutol and five months of Isoniazid, Rifampicin and Ethambutol. Among TB patients, a subset of patients who were at high risk to have MDR-TB (presumptive MDR-TB patients) underwent genotypic drug susceptibility testing (DST). These included patients previously treated for TB, patients with a TB-HIV co-infection, patients who upon follow up during TB treatment were smear-positive and contacts of a confirmed MDR-TB patient.

Project *Axshya*: Project *Axshya* is implemented in India by the South-East Asia office of the International Union against Tuberculosis and Lung Disease (The Union) to enhance the reach and visibility of RNTCP services among marginalised and vulnerable populations and to mitigate the impact of TB on the country (see Box for criteria for marginalised and vulnerable populations). *Axshya SAMVAD* ( *SAMVAD* is an acronym for sensitization and advocacy in marginalised and vulnerable areas of the district) is the ACF strategy under the project. The word ‘ *SAMVAD’* in Sanskrit language means ‘conversation’. In 2016–17, the project covered 285 districts spread across 19 states.

Box. Criteria used for marginalised and vulnerable populations in districts under Project Axshya*, India (2016–17).

1. Slums

2. Tribal areas

3. Marginalised communities as per the constitution of India

4. In pockets where occupational lung diseases are high

5. In pockets where there is high risk of acquiring TB like; stone crushing/mining/weaving industry/unorganized labour (construction workers etc)/homeless people

6. In pockets reported to have high HIV/ AIDS burden

7. In areas or communities where incidence of TB is high

8. Among household contacts of smear-positive pulmonary TB patients

9. Prisons

TB – tuberculosis; HIV – human immunodeficiency virus; AIDS – acquired immunodeficiency syndrome; *Project Axshya –implemented by The Union, South East Asia office, New Delhi, India, across 285 districts of India, to enhance the reach and visibility of national TB programme services among marginalised and vulnerable populations and to mitigate the impact of TB on the country. Axshya in Sanskrit means ‘free of TB’.

*Axshya SAMVAD* study: This study was conducted among new smear-positive pulmonary TB patients to determine the effectiveness of *Axshya SAMVAD* on diagnosis and treatment initiation delays, costs due to TB diagnosis and treatment outcomes ^6, 7^. We included all new smear-positive pulmonary TB patients from marginalised and vulnerable populations that were detected through ACF and registered under the programme in the 18 randomly sampled *Axshya* districts (simple random sampling) during March 2016 to February 2017. Every month in the same districts, we randomly sampled an equal number of new smear-positive pulmonary TB patients registered through PCF from marginalised and vulnerable populations (simple random sampling) ^6, 7^. Random numbers for simple random sampling were generated using Microsoft Excel.

### Data collection

Under *Axshya SAMVAD* study, we collected data for each study participant through record review (age, sex, ACF/PCF status, residence (urban/rural), distance of residence from microscopy centre, sputum smear grade, weight, diabetes status and HIV status) and patient interviews at their residence. Patient interviews were set up during the review of the participant’s record. Before starting the patient interviews, we enquired about their past history of TB treatment for at least one month either from the public or private sector. Those with a past history of treatment were excluded from the *Axshya SAMVAD* study and referred to the programme for appropriate management. These constitute ‘misclassification’ for the purpose of present analysis.

### Data analysis

We double entered and validated the data using EpiData Entry software ^9^ (version 3.1, EpiData Association, Odense Denmark). We analysed the data using STATA (version 12.1, copyright 1985–2011 StataCorp LP USA) ^10^. We used frequency and proportions (0.95 confidence intervals (CI)) to summarise (infer) the extent of misclassification. Adjusted analysis was done using log binomial regression to determine the factors associated with misclassification. Variables collected during record review (age, sex, ACF/PCF status, residence (urban/rural), distance of residence from microscopy centre and sputum smear grade) were included in the adjusted analysis. Baseline weight was missing in two-fifths of patients; baseline diabetes status was missing for more than three-fifths and HIV status was missing for two-fifths. Hence, we excluded them from the adjusted analysis. The association was summarized (inferred) using adjusted prevalence ratios (95% CIs).

### Ethics

The *Axshya SAMVAD* study was approved by the Ethics Advisory Group of The Union, Paris, France (EAG number 15/15, dated 28 September 2015). We conducted the study after receiving approvals from the State Tuberculosis Officers in the respective states (18 randomly sampled *Axshya* districts belonged to seven states). We obtained written informed consent for participation from all the study participants.

## Results

**Figure 1.**
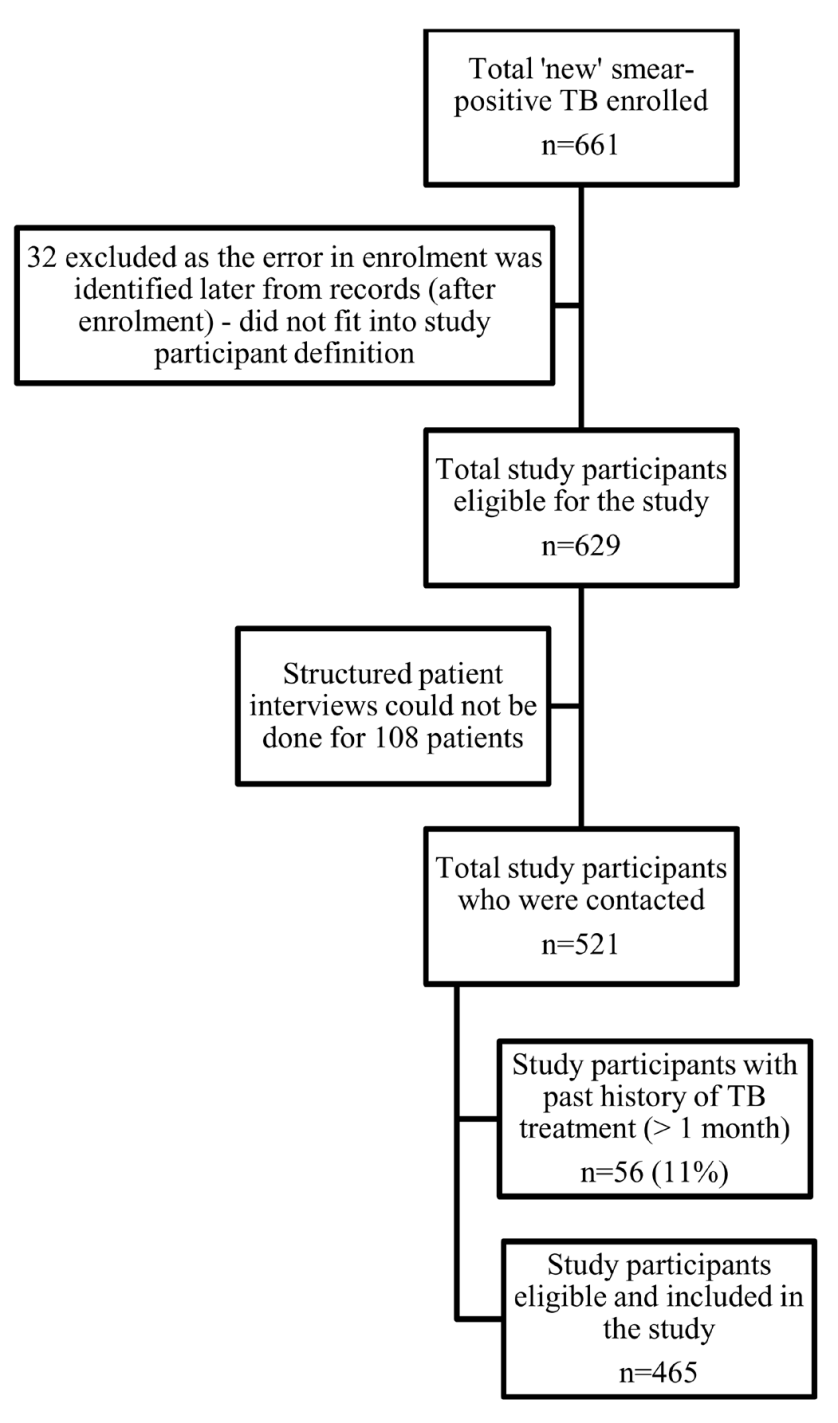
Flow chart depicting the misclassification of people (≥ 15 y) with ‘previously treated’ smear-positive pulmonary TB as ‘new’ among the study participants enrolled in the *Axshya SAMVAD* study across 18 randomly sampled *Axshya* districts in India, March 2016-February 2017*. TB – tuberculosis; SAMVAD – sensitization and advocacy in marginalised and vulnerable areas of the district; Axshya SAMVAD – an active case- finding strategy under project Axshya, implemented by The Union, South East Asia office, New Delhi, India, across 285 districts of India. *registered under programme between March 2016 and February 2017 for treatment after classification as ‘new’.

Figure 1 depicts the misclassification of ‘previously treated’ smear-positive pulmonary TB patients as ‘new’. A total of 629 newly registered smear-positive pulmonary TB patients were enrolled for the *Axshya SAMVAD* study. We couldn’t contact 108 (17%) for interview as patients were not available at their residence during the visit (a maximum of two visits were made).

Of the 521 interviewed, 56 [10.8% (95% CI: 8.4%, 13.7%)] had a past history of TB treatment (public or private) for at least a month: 12.7% (34/268) among the ACF group and 8.7% (22/253) among the PCF group (p=0.18). No factors were found to be significantly associated with misclassification ( Table 1). Patients belonging to rural areas had higher prevalence of misclassification when compared to urban areas (12% vs 2%), but this difference was not statistically significant probably due to small sample size.

**Table 1.** Factors associated with the misclassification of ‘previously treated’ smear-positive pulmonary TB as ‘new’ among the new smear-positive pulmonary TB patients (≥ 15 y) in the *Axshya SAMVAD* study across 18 randomly sampled *Axshya* districts in India, March 2016-February 2017 *.

Variable	Total	Misclassification		PR (95% CI)	aPR ^@^ (95% CI)
	N	n	(%)		
Total	521 **	56	(11)	-	-
Exposure					
*Axshya SAMVAD*	268	34	(13)	1.5 (0.9, 2.4)	1.3 (0.7, 2.1)
Passive case finding	253	22	(9)	Ref	Ref
Age categories in years					
15–44	276	25	(9)	Ref	Ref
45–64	185	22	(12)	1.3 (0.8, 2.3)	1.1 (0.6, 1.9)
≥65	59	9	(15)	1.7 (0.8, 3.4)	1.5 (0.7, 3.1)
Missing	1	0	(0)	-	-
Sex					
Male	346	39	(11)	1.2 (0.7, 2.0)	1.3 (0.7, 2.2)
Female	174	17	(10)	Ref	Ref
Missing	1	0	(0)	-	-
Residence					
Urban	59	1	(2)	Ref	Ref
Rural	457	55	(12)	7.1 (1.0, 50.4)	6.4 (0.9, 48.2)
Missing	5	0	(0)	-	-
Distance from DMC in km					
≤5	128	10	(8)	Ref	Ref
6–10	161	17	(11)	1.4 (0.6, 2.8)	1.0 (0.5, 2.0)
11–15	118	11	(9)	1.2 (0.5, 2.7)	0.8 (0.4, 1.9)
>15	113	17	(15)	1.9 (0.9, 4.0)	1.4 (0.7, 2.9)
Missing	1	1	(100)	-	-
Sputum smear grading					
3+	90	7	(8)	Ref	Ref
1+/2+	413	48	(12)	1.5 (0.7, 3.2)	2.4 (0.3, 16.2)
Positive not quantified	18	1	(6)	-	-

*TB – tuberculosis; SAMVAD – sensitization and advocacy in marginalised and vulnerable areas of the district; Axshya SAMVAD – an active case-finding strategy under project Axshya implemented by The Union, South East Asia office, New Delhi, India, across 285 districts of India; aPR – adjusted prevalence ratio; CI – confidence interval*. *registered under programme between March 2016 and February 2017 for treatment after classification as ‘new’; **Total 661 were enrolled, 32 were later excluded as they did not fit the operational definition of study participant based on information obtained from record review. Among 629 eligible for patient interviews, 521 study participants could be contacted; ^**@**^log binomial regression.

## Discussion

### Key findings

About one in ten ‘new’ TB patients had a past history of TB treatment. This misclassification meant that these patients received the wrong treatment regimen as per the national guidelines at the time. This is similar to the RNTCP report of 2018 ^3^ and previous documentation in 2007 ^5^. The misclassification among new smear-positive TB patients was two times higher than the 4.5% reported from Malawi in 2000 ^11^.

One possible reason for this might be a lack of attention on the part of the medical officer to enquire for previous history of TB before starting treatment. Ambiguity in classification when there was a large gap between previous and current treatment, absence of treatment records and patients’ reluctance to disclose previous treatment details due to possible stigma (fear of being seen as a ‘problem patient’) could be the other reasons ^5^.

### Limitations

This study has some limitations. First, this programmatically relevant finding was incidental and part of a larger study ( *Axshya SAMVAD* study) and hence, we did not systematically record the details of past TB treatment (when, duration of treatment, whether under programme or in private sector) and the reasons for misclassification. Secondly, as patients with misclassification were excluded from the *Axshya SAMVAD* study, we do not know what happened to them, including their treatment outcomes. Thirdly, we did not include smear-negative pulmonary TB and extrapulmonary TB patients as they were not part of the *Axshya SAMVAD* study. In Malawi (2000) ^11^, they had a higher risk of misclassification when compared to smear-positive pulmonary TB patients. Finally, non-response was a limitation. However, in a best-case scenario (assuming all 108 non-responders did not have previous history of TB treatment), the proportion of misclassification would have been 8.9% (56/629) which is still programmatically significant.

### Implications for the TB programme

Limitations notwithstanding, our study has programme implications. Of the new smear-positive pulmonary TB patients registered in India in 2016, 21% had an unfavourable outcome ^3^. Some of these unfavourable outcomes can be explained by wrong management – patients getting an inferior treatment regimen (previously treated patients being treated with a regimen meant for new cases) and missing an opportunity for drug susceptibility testing (as previously treated patients were eligible for DST at the time). Inferior regimen might have also contributed to amplification of resistance in those who may have primary or acquired drug resistance (from prior treatment) and MDR-TB. This has been happening for over 10 years so one can see why India now faces the serious problem of drug resistant TB.

India has recently adopted the World Health Organization (WHO) recommendation that the category II regimen (for ‘previously treated’ patients) ‘ *should no longer be prescribed and drug susceptibility testing should be conducted to inform the choice of treatment regimen*’ ^12^. To make this a reality, India now recommends universal DST, meaning all diagnosed TB patients are eligible for testing via the Xpert MTB/RIF assay ^® ^(Cepheid Sunnyvale USA) followed by first-line (if rifampicin susceptible) or second-line line probe assay (if rifampicin resistant) ^4, 13^. This further means that both new and previously treated patients are treated with the same regimen ^4, 8, 14^. Hence, in the present scenario, the impact of misclassification on individual patient management is minimal. This was not the case at the time of conduct of this study. Despite these developments, we think asking for previous treatment history is still relevant for two reasons. First, the information on the proportion of previously treated patients is epidemiologically an important piece of information and is regularly reported to the WHO for monitoring the global TB epidemic. Second, the universal DST is not a reality in every part of the country and in such instances, prioritizing previously treated patients for DST is a better strategy, given the higher prevalence of drug-resistant TB among them.

### Recommendations

Our findings were based on patients from marginalised and vulnerable populations and this limits our generalisability to TB patients registered from the general population. The programme should consider replicating similar studies among patients from the general population with a possible sub-group to look for rural-urban differences.

Since 2017, the revised laboratory register at the level of designated microscopy centres under the RNTCP (one per 50 000 to 100 000 population) also captures this information of previous treatment ^8^. RNTCP staff needs to be re-sensitized to “ask” for previous history of TB treatment. Hence, complete filling of the revised laboratory register at microscopy centres should be closely monitored by the programme and future operational research should focus on this.

Systematic qualitative enquiry is recommended to understand the ‘why’ (why does it happen) and ‘how’ (how can it be addressed) of misclassification. In the national case-based TB notification software ( *NIKSHAY*), record linkage and deduplication using key attributes may be considered to identify repeat notification of the same person separated by a time period.

## Conclusions

This study demonstrated that ‘previously treated’ patients were being missed and were being registered as ‘new’ patients under the RNTCP in India. Corrective measures need to be implemented, followed by monitoring any change in the proportion of ‘previously treated’ patients among all registered patients treated under the programme at national level.

## Data availability

### Underlying data

figshare: Underlying data. https://dx.doi.org/10.6084/m9.figshare.7756688 ^15^

Data are available under the terms of the Creative Commons Zero "No rights reserved" data waiver (CC0 1.0 Public domain dedication).

### Extended data

figshare: Questionnaire Axshya SAMVAD study. https://dx.doi.org/10.6084/m9.figshare.7768589 ^16^

This project contains the following extended data:

- S2 Annex.pdf (Part I of the questionnaire – record review)
- S3 Annex.pdf (Part II of the questionnaire – patient interview)

Data are available under the terms of the Creative Commons Zero "No rights reserved" data waiver (CC0 1.0 Public domain dedication).

## Consent

Written informed consent for publication of the patients’ details was obtained from the patients.
