# Supplementary material for: Are we missing ‘previously treated’ smear-positive pulmonary tuberculosis under programme settings in India? A cross-sectional study
Source: F1000Res. 2019 May 16;8:338. Originally published 2019 Mar 26. [Version 2] doi: 10.12688/f1000research.18353.2 (PMC6600860; doi:10.12688/f1000research.18353.2)
Supplement: Peer review history

# Peer review: Reviewer response for version 2

**DOI:** 10.5256/f1000research.21019.r49370

**Published:** 2019-06-27

**Authors:** Banurekha Velayutham

**Affiliations:** 1 ICMR–National Institute for Research in Tuberculosis (ICMR-NIRT), Chennai, Tamil Nadu, India

The authors have attempted to quantify the magnitude of misclassification among new smear positive pulmonary TB patients. In the current scenario of universal DST and removal of Category II regimen, this information may not of much value. Nevertheless, the 11% misclassification emphasizes the importance of appropriate history taking which could benefit patient management. Though new and previously treated patients are treated with the same 6-month daily regimen if found to be drug sensitive the distinction based on previous treatment would be important to analyse the response to treatment in future.

 The major limitation of this study is that it was not planned but rather incidental as mentioned by the authors. Hence, the reasons for such misclassification from patient and provider perspective which could have added more value to the study is lacking. The findings are worth communicating as a Brief communication article. Some clarifications are requested on the following: 

1. What was the time interval between registration for treatment and the patient interview
2. The rationale for considering distance from DMC as a factor for misclassification

I confirm that I have read this submission and believe that I have an appropriate level of expertise to confirm that it is of an acceptable scientific standard, however I have significant reservations, as outlined above.

# Peer review: Reviewer response for version 2

**DOI:** 10.5256/f1000research.21019.r48633

**Published:** 2019-05-20

**Authors:** Sachin R. Atre

**Affiliations:** 1 Center for Clinical Global Health Education (CCGHE), Johns Hopkins University, Baltimore, MD, USA; 2 Dr. D.Y. Patil Medical College, Hospital and Research Centre Pune, Pimpri-Chinchwad, India

I feel many of my points are not addressed by the authors except a few minor changes.

I confirm that I have read this submission and believe that I have an appropriate level of expertise to confirm that it is of an acceptable scientific standard, however I have significant reservations, as outlined above.

# Peer review: Reviewer response for version 2

**DOI:** 10.5256/f1000research.21019.r48632

**Published:** 2019-05-16

**Authors:** Otavio T. Ranzani

**Affiliations:** 1 Pulmonary Division, Heart Institute (InCor), Hospital das Clinicas (HCFMUSP), Faculdade de Medicina, University of São Paulo, São Paulo, Brazil; 2 Barcelona Institute for Global Health (ISGlobal), Barcelona, Spain

The authors addressed all my comments.

I confirm that I have read this submission and believe that I have an appropriate level of expertise to confirm that it is of an acceptable scientific standard.

# Peer review: Reviewer response for version 1

**DOI:** 10.5256/f1000research.20077.r46257

**Published:** 2019-04-12

**Authors:** Otavio T. Ranzani

**Affiliations:** 1 Pulmonary Division, Heart Institute (InCor), Hospital das Clinicas (HCFMUSP), Faculdade de Medicina, University of São Paulo, São Paulo, Brazil; 2 Barcelona Institute for Global Health (ISGlobal), Barcelona, Spain

The authors conducted a secondary analysis on the data originated from the *Axshya SAMVAD* study aiming to describe the proportion of patients with smear-positive pulmonary tuberculosis labelled as "new" (regarding antibiotic treatment), but that actually have had tuberculosis before. The research question is of public health importance, it is not widely reported in the literature, and makes this short article interesting to the literature. The authors should be commended. I have some comments below aiming to improve the manuscript.

1. The authors should clarify to the reader on abstract that "new" refers to previous TB treatment for at least 30 consecutive days.
2. The authors should revise the entire manuscript, particularly Abstract and Introduction, to clarify about what "missclassification" refers to. Misclassification is an epidemiological term, but it can refer to different variables depending on the context. And during the read, it became clear only after going to the results section. Example: (abstract) "reported 11% misclassification among ‘new’ patients". Maybe the authors could rephrase as:"reported that 11% of patients with tuberculosis were misclassified as new patients regarding previous treatment history" or "reported that 11% of patients with tuberculosis were misclassified as new patients despite their previous TB treatment" or "reported that 11% of patients with tuberculosis were misclassified as new patients despite previously treated"
3. Regarding the inclusion criteria, did it only include adults?
4. Regarding Table 1: Why did you categorize age? And why in only 3 categories merging different age profile? For instance, the range 15-44 covers different population. You might have loose power and contrast. You should use age as continuous and/or open the category 15-44. Do the authors have converge problems with the log-binomial model? The authors should re-phrase their statements on the prevalence ratio associated factors on Table 1. For instance, it is clear that Residence (rural/urban) is an important factor, with a big point estimate, but wide confidence interval (6.4 (0.9, 48.2)). Likely with a bigger sample size and/or higher number of events, you would have better precision and would achieve "significancy" or "no include 1". 
5. How is it possible to have such low prevalence of positive HIV status in a vulnerable population? How was the HIV testing coverage?
6. The discussion section must be improved: Although recommended and ideal, DST will not be available for all. Even if available, the previous history treatment is fundamental. The authors should propose solutions for the problem, such as linkage deduplication, linked records form the national system, etc. Please, better discuss the potential selection bias and representative of the included population regarding India population.

Minor:

1. Maybe better to use the notation of 95% CI rather than 0.95
2. Figure 1. I think the first box at left has a wrong "the" : as AN error
3. Change gender to sex

I confirm that I have read this submission and believe that I have an appropriate level of expertise to confirm that it is of an acceptable scientific standard, however I have significant reservations, as outlined above.

# Response

**Published:** 2019-05-07

**Authors:** Hemant Deepak Shewade

**Affiliations:** International Union Against Tuberculosis and Lung Disease, India

**REVIEWER #2**

**Otavio T. Ranzani, University of São Paulo, Brazil; Barcelona Institute for Global Health (ISGlobal), Spain**

REVIEWER COMMENT

The authors conducted a secondary analysis on the data originated from the Axshya SAMVAD study aiming to describe the proportion of patients with smear-positive pulmonary tuberculosis labelled as "new" (regarding antibiotic treatment), but that actually have had tuberculosis before. The research question is of public health importance, it is not widely reported in the literature, and makes this short article interesting to the literature. The authors should be commended. I have some comments below aiming to improve the manuscript.

AUTHOR RESPONSE

Thank you very much for the constructive comments.

REVIEWER COMMENT

The authors should clarify to the reader on abstract that "new" refers to previous TB treatment for at least 30 consecutive days.

AUTHOR RESPONSE

We are not clear here what the reviewer intends to mean. ‘New’ refers patients who have not been previously treated for TB (at least for one month).

REVIEWER COMMENT

The authors should revise the entire manuscript, particularly Abstract and Introduction, to clarify about what "misclassification" refers to. Misclassification is an epidemiological term, but it can refer to different variables depending on the context. And during the read, it became clear only after going to the results section. Example: (abstract) "reported 11% misclassification among ‘new’ patients". Maybe the authors could rephrase as: "reported that 11% of patients with tuberculosis were misclassified as new patients regarding previous treatment history" or "reported that 11% of patients with tuberculosis were misclassified as new patients despite their previous TB treatment" or "reported that 11% of patients with tuberculosis were misclassified as new patients despite previously treated"

AUTHOR RESPONSE

Thank you for the comment. In the revised manuscript, both at beginning of abstract and main text introduction, we have clarified as to what we mean by ‘misclassification’ (reproduced below)

Abstract

“In 2007, a field observation from India reported 11% misclassification among ‘new’ patients registered under the revised national tuberculosis (TB) control programme. Ten years down the line, it is important to know what proportion of newly registered patients has a past history of TB treatment for at least one month (henceforth called as ‘misclassification’).”

Introduction (main text)

“This provided us with a unique opportunity to document the proportion of newly registered smear-positive pulmonary TB patients that had previous history of TB treatment and were therefore misclassified (henceforth called as ‘misclassification’).”

REVIEWER COMMENT

Regarding the inclusion criteria, did it only include adults?

AUTHOR RESPONSE

Thank you for pointing this out. Yes it included only adults (≥ 15 y). We have clarified this under study population and titles of tables and figures.

REVIEWER COMMENT

Regarding Table 1: Why did you categorize age? And why in only 3 categories merging different age profile? For instance, the range 15-44 covers different population. You might have loose power and contrast. You should use age as continuous and/or open the category 15-44. Do the authors have converge problems with the log-binomial model? The authors should re-phrase their statements on the prevalence ratio associated factors on Table 1. For instance, it is clear that Residence (rural/urban) is an important factor, with a big point estimate, but wide confidence interval (6.4 (0.9, 48.2)). Likely with a bigger sample size and/or higher number of events, you would have better precision and would achieve "significancy" or "no include 1".

AUTHOR RESPONSE

Thank you for the comment. We categorized aged, because we wanted to explore whether misclassification was significantly higher among certain meaningful sub-groups. Regarding opening up the category 15-44 y, we decided not to do this as there are only 25 events of interest (=misclassification) in this sub-group. If we further divide this sub-group, the event of interest could get reduced and this would have further widened the 95% CI for the adjusted prevalence ratios.

As per reviewer comments, we have added a line under results narrative in the revised manuscript to discuss at adjusted prevalence ratio for the variable ‘residence’ (reproduced below).

“Patients belonging to rural areas had higher prevalence of misclassification when compared to urban areas (12% vs 2%), but this difference was not statistically significant probably due to small sample size.”

REVIEWER COMMENT

How is it possible to have such low prevalence of positive HIV status in a vulnerable population? How was the HIV testing coverage?

AUTHOR RESPONSE

The HIV percentage among all TB patients in India is around 3%. In our study, HIV status was missing in records for two-fifth patients and among those whose test results were recorded (n=288), one was positive.

REVIEWER COMMENT

The discussion section must be improved: Although recommended and ideal, DST will not be available for all. Even if available, the previous history treatment is fundamental. The authors should propose solutions for the problem, such as linkage deduplication, linked records form the national system, etc. Please, better discuss the potential selection bias and representative of the included population regarding India population.

AUTHOR RESPONSE

Thank you very much. We have reviewed and revised the discussion section as per the comments.

We agree that DST may not be available for all. Hence, in the “Implications for the TB programme” sub-section under discussion section we have included this this. We are reproducing it below:

“Second, the universal DST is not a reality in every part of the country and in such instances, prioritizing previously treated patients for DST is a better strategy, given the higher prevalence of drug-resistant TB among them.”

Regarding DST availability, we are reproducing the relevant discussion lines below

“Hence, in the present scenario, the impact of misclassification on individual patient management is minimal. This was not the case at the time of conduct of this study. Despite these developments, we think asking for previous treatment history is still relevant for two reasons. First, the information on the proportion of previously treated patients is epidemiologically an important piece of information and is regularly reported to the WHO for monitoring the global TB epidemic. Second, the universal DST is not a reality in every part of the country and in such instances, prioritizing previously treated patients for DST is a better strategy, given the higher prevalence of drug-resistant TB among them” (second paragraph under ‘implications for TB programme’ sub-section of discussion)

Regarding potential selection bias and representativeness, we are reproducing the relevant discussion lines below:

“Our findings were based on patients from marginalised and vulnerable populations and this limits our generalisability to TB patients registered from the general population. The programme should consider replicating similar studies among patients from the general population with a possible sub-group to look for rural-urban differences.” (First paragraph under ‘recommendations’ sub-section of discussion)

Regarding proposing solutions, we have revised it as per reviewer suggestions under recommendations subsection of discussion (reproducing below)

“Since 2017, the revised laboratory register at the level of designated microscopy centres under the RNTCP (one per 50 000 to 100 000 population) also captures this information of previous treatment ^8^ . RNTCP staff needs to be re-sensitized to “ask” for previous history of TB treatment.  Hence, complete filling of the revised laboratory register at microscopy centres should be closely monitored by the programme and future operational research should focus on this.

Systematic qualitative enquiry is recommended to understand the ‘why’ (why does it happen) and ‘how’ (how can it be addressed) of misclassification. In the national case-based TB notification software ( *NIKSHAY*), record linkage and deduplication using key attributes may be considered to identify repeat notification of the same person separated by a time period.”

REVIEWER COMMENT

Minor:

Maybe better to use the notation of 95% CI rather than 0.95

AUTHOR RESPONSE

We have revised throughout the manuscript as per reviewer suggestion

REVIEWER COMMENT

Figure 1. I think the first box at left has a wrong "the" : as AN error

AUTHOR RESPONSE

We are not clear as to what the reviewer means here. We did not find this error in the first left box of figure 1. However, we re-checked figure 1 and found it no grammatical errors.

REVIEWER COMMENT

Change gender to sex

AUTHOR RESPONSE

We have revised throughout the manuscript as per reviewer suggestion

# Peer review: Reviewer response for version 1

**DOI:** 10.5256/f1000research.20077.r46256

**Published:** 2019-04-09

**Authors:** Sachin R. Atre

**Affiliations:** 1 Center for Clinical Global Health Education (CCGHE), Johns Hopkins University, Baltimore, MD, USA; 2 Dr. D.Y. Patil Medical College, Hospital and Research Centre Pune, Pimpri-Chinchwad, India

The manuscript addresses one of the crucial problems with in India’s Revised National TB Control Program. Interestingly, the study is based on our prior study (in which I was a lead author) in 2007, which was conducted in Mumbai and rural areas of Pune district. Given the research context and the data, I feel this manuscript should go as a brief communication or notes from the field rather than a full original research article. It does not make any novel contribution, but just confirms the earlier research finding. Even to make it a brief communication, I feel some points need to be provocatively addressed. My comments are as below.

1. Authors have provided details of their larger study, of which the current study is only a part. I feel that level of details is unnecessary here. On the other hand, unfortunately despite having a large team (as seen from the long list of authors), they did not do in-depth inquiry into the reasons for an erroneous categorization of cases, which is actually the main aim of the manuscript. They just mentioned the same reasons for erroneous categorization by providing a reference to our 2007 article without making any new contribution. Identifying the most prominent reasons would have been helpful to identify as a focus area for the policy makers to make some action plan for operational implementation of the program. In my opinion, there is not much substance to publish it as an original research article.
2. Authors nowhere discussed the major implication of their observation that even after 10 years, there remains a big disconnect between the operational research and the program implementation, which is really unfortunate. This finding has another major implication that because of erroneous categorization, previously treated cases are being treated with first-line regimen, which results in amplification of resistance in those cases who may have primary or acquired drug resistance (from prior treatment) and/MDR-TB. This has been happening for over 10 years so one can see why India now faces the serious problem of drug resistant TB.
3. I am not convinced with the factor analysis in Table 1 because the detailed inquiry was not made into reasons for misclassification/erroneous categorization which should have been the main focus.
4. Authors state in discussion that as per the WHO recommendation, universal DST will be done for all TB cases. This is an ideal situation. The scale up of GeneXpert even remains questionable. The WHO global report 2018 showed that only 40% of TB cases in India were subjected to GeneXert in 2017. There are problems of shortage of cartridges, falcon tubes, power outages etc. Given the glacial speed of translation of findings from operational research in the actual RNTCP implementation, India’s claim of TB elimination by 2025 looks really questionable.

I confirm that I have read this submission and believe that I have an appropriate level of expertise to confirm that it is of an acceptable scientific standard, however I have significant reservations, as outlined above.

# Response

**Published:** 2019-05-07

**Authors:** Hemant Deepak Shewade

**Affiliations:** International Union Against Tuberculosis and Lung Disease, India

**REVIEWER #1**

**Sachin R. Atre, Johns Hopkins University, USA; Dr. D.Y. Patil Medical College, Hospital and Research Centre Pune, India**

REVIEWER COMMENT

The manuscript addresses one of the crucial problems with in India’s Revised National TB Control Program. Interestingly, the study is based on our prior study (in which I was a lead author) in 2007, which was conducted in Mumbai and rural areas of Pune district. Given the research context and the data, I feel this manuscript should go as a brief communication or notes from the field rather than a full original research article. It does not make any novel contribution, but just confirms the earlier research finding. Even to make it a brief communication, I feel some points need to be provocatively addressed. My comments are as below.

AUTHOR RESPONSE

Thank you for the comment. F1000Research does not have the article types as suggested: brief communication or notes from the field. ‘Research notes’ is the closest option, but here too there is no specific word count mentioned. Considering our manuscript is around 1800 words only, we are fine if F1000Research agrees to consider this as a research note.

We agree the findings are not novel, but are timely considering 10 years down the line, the issue remains as it is and this is important to highlight.

We have addressed your comments, details below.

REVIEWER COMMENT

Authors have provided details of their larger study, of which the current study is only a part. I feel that level of details is unnecessary here. On the other hand, unfortunately despite having a large team (as seen from the long list of authors), they did not do in-depth inquiry into the reasons for an erroneous categorization of cases, which is actually the main aim of the manuscript. They just mentioned the same reasons for erroneous categorization by providing a reference to our 2007 article without making any new contribution. Identifying the most prominent reasons would have been helpful to identify as a focus area for the policy makers to make some action plan for operational implementation of the program. In my opinion, there is not much substance to publish it as an original research article.

AUTHOR RESPONSE

The actual study was done across 18 randomly spread districts of the country (Axshya SAMVAD study – Axshya SAMVAD is an ACF strategy for detecting TB and is implemented by The Union South East Asia). As mentioned, the findings were accidental (not intended) and we thought it is important to report this and look for differences in rates of misclassification across various patient sub-groups.

The Axshya SAMVAD study was conducted by project Axshya staff in operational setting without any additional funding for the study itself. The research team in the field (project Axshya staff) were not trained and did not have the required capacity to conduct qualitative research. Hence, we could not go in-depth into the reasons. But, one key point is that our study was conducted in randomly selected 18 districts of the country and hence, the findings are representative.

We agree the findings are not novel, but are timely considering 10 years down the line, the issue remains as it is and this is important to highlight.

Considering our manuscript is around 1800 words only, we are fine if F1000Research agrees to consider this as a research note.

REVIEWER COMMENT

Authors nowhere discussed the major implication of their observation that even after 10 years, there remains a big disconnect between the operational research and the program implementation, which is really unfortunate. This finding has another major implication that because of erroneous categorization, previously treated cases are being treated with first-line regimen, which results in amplification of resistance in those cases who may have primary or acquired drug resistance (from prior treatment) and/MDR-TB. This has been happening for over 10 years so one can see why India now faces the serious problem of drug resistant TB.

AUTHOR RESPONSE

Thank you very much for your comment. We agree with the reviewer. We have included the above point in the revised manuscript as suggested by the reviewer. We have discussed the implications in the first paragraph of ‘Implications for the TB programme’ in the revised manuscript (reproduced below):

“Limitations notwithstanding, our study has programme implications. Of the new smear-positive pulmonary TB patients registered in India in 2016, 21% had an unfavourable outcome ^3^ . Some of these unfavourable outcomes can be explained by wrong management – patients getting an inferior treatment regimen (previously treated patients being treated with a regimen meant for new cases) and missing an opportunity for drug susceptibility testing (as previously treated patients were eligible for DST at the time). Inferior regimen might have also contributed to amplification of resistance in those who may have primary or acquired drug resistance (from prior treatment) and MDR-TB. This has been happening for over 10 years so one can see why India now faces the serious problem of drug resistant TB.”

REVIEWER COMMENT

I am not convinced with the factor analysis in Table 1 because the detailed inquiry was not made into reasons for misclassification/erroneous categorization which should have been the main focus.

AUTHOR RESPONSE

Thank you for the comment. In Table 1, we are quantitatively looking for patient sub-groups who have higher prevalence of misclassification. Though not statistically significant, programmatically significant differences were observed in misclassification in urban and rural areas (12%  in rural and 2% in urban areas). We agree that we have not looked into the ‘why’ and ‘how’ of misclassification (limitations). But, based on our programme experiences made some useful recommendations in the revised manuscript (reproduced below)

“Our findings were based on patients from marginalised and vulnerable populations and this limits our generalisability to TB patients registered from the general population. The programme should consider replicating similar studies among patients from the general population with a possible sub-group to look for rural-urban differences.

Since 2017, the revised laboratory register at the level of designated microscopy centres under the RNTCP (one per 50 000 to 100 000 population) also captures this information of previous treatment ^8^ . RNTCP staff needs to be re-sensitized to “ask” for previous history of TB treatment.  Hence, complete filling of the revised laboratory register at microscopy centres should be closely monitored by the programme and future operational research should focus on this.

Systematic qualitative enquiry is recommended to understand the ‘why’ (why does it happen) and ‘how’ (how can it be addressed) of misclassification. In the national case-based TB notification software ( *NIKSHAY*), record linkage and deduplication using key attributes may be considered to identify repeat notification of the same person separated by a time period.”

REVIEWER COMMENT

Authors state in discussion that as per the WHO recommendation, universal DST will be done for all TB cases. This is an ideal situation. The scale up of GeneXpert even remains questionable. The WHO global report 2018 showed that only 40% of TB cases in India were subjected to GeneXert in 2017. There are problems of shortage of cartridges, falcon tubes, power outages etc. Given the glacial speed of translation of findings from operational research in the actual RNTCP implementation, India’s claim of TB elimination by 2025 looks really questionable.

AUTHOR RESPONSE

Thank you for the comment. We agree with the reviewer. We agree that DST for all TB patients is an ideal situation. Therefore, we have also stated that in a non-ideal situation (DST not possible for all TB patients), DST may be focussed on those with previous history of TB treatment. Therefore, correct classification as new or previously treated is still relevant. We have mentioned this in the last three lines of the section ‘implications for the TB programme’ (reproduced below):

“Second, the universal DST is not a reality in every part of the country and in such instances, prioritizing previously treated patients for DST is a better strategy, given the higher prevalence of drug-resistant TB among them.”
